# Return to Work in Breast Cancer Patients following an Interdisciplinary Rehabilitation Program in a Community-Based Cancer Rehabilitation Center: A Pilot Study

**DOI:** 10.3390/healthcare12070805

**Published:** 2024-04-07

**Authors:** Matthew Rong Jie Tay, Chin Jung Wong, Hui Zhen Aw

**Affiliations:** 1 Department of Rehabilitation Medicine, Tan Tock Seng Hospital, 11 Jalan Tan Tock Seng, Singapore 308433, Singapore; 2 Singapore Cancer Society Rehabilitation Centre, 30 Hospital Boulevard, National Cancer Centre Singapore Building, Singapore 168583, Singapore

**Keywords:** return to work, cancer rehabilitation, breast neoplasms, fatigue, rehabilitation centers

## Abstract

Despite curative treatment and discharge from acute hospital settings, breast cancer patients often have cancer- and treatment-related morbidity which impairs them from returning to work. Hence, the role of community-based return to work rehabilitation programs is important to help these patients transition back to work. This was a retrospective cohort study involving patients with breast cancer conducted at a community-based cancer rehabilitation center. Patients were involved in an interdisciplinary vocational rehabilitation program involving physiatrists, occupational therapists, physiotherapists and social workers. We recruited 63 patients for this study cohort, with 46 (73.0%) patients ≤ 60 years old. After undergoing the rehabilitation program, there were 37 (58.7%) participants who successfully returned to work. These participants returned to work at either within 6 months (27.0%), 12 months (29.7%) or 24 months (43.2%) after enrollment into the program, with a majority enrolling in white collar jobs. Multivariate regression analysis revealed that significant negative factors for return to work were advanced stage of cancer (*p* = 0.004), along with clinically significant fatigue, measured on the Brief Fatigue Inventory (*p* < 0.001). However, perceived work ability (*p* = 0.020) was found to be a positive factor.

## 1. Introduction

Successful breast cancer treatment has led to an increased survival rate and a growing number of people living with a previous cancer diagnosis [1]. Despite progress in breast cancer treatment, the majority of patients experience morbidity, either from the disease itself or treatment toxicity, which has negative impacts on all aspects of their lives [2,3]. Many of these patients are of working age [4], with a substantial proportion of cancer patients being younger than 65 years [5]. Patients often express a desire to return to work (RTW) as being an important aspect of cancer survivorship, and re-engaging in paid employment can provide a sense of productivity and recovery, along with fulfilling financial needs [6]. However, impaired physical functioning amongst patients often results in an inability to work or limitation in their ability to work, with unemployment or reduced employed hours commonly being reported [7,8]. This has resulted in cancer patients being more likely to be unemployed than healthy controls, with a systemic review revealing that more than 33% of cancer patients were unemployed, compared to 15% in healthy controls [4]. Additionally, a higher reported unemployment rate has been reported in patients with breast, gastrointestinal and female reproductive organ cancers compared with other cancers [4].

Return to work has profound implications across economic, psychological, social and educational domains for breast cancer patients. By re-entering the workforce, survivors can mitigate the financial burdens associated with cancer treatment, including medical expenses and lost or diminished earning capacity [9]. Returning to work can also improve the psychological well-being of breast cancer patients and increase social contact at work. Impaired RTW has been significantly associated with depressive symptoms, with temporal patterns of general quality of life (QoL) being significantly worse among women with no RTW compared with those working [10]. The process of returning to work can give patients a sense of identity and purpose, and can be seen as a means for returning to normality [11].

Although there is strong evidence of the benefits of cancer rehabilitation on physical function [12,13], there have been limited studies on the effects of community-based cancer rehabilitation programs on RTW outcomes in Asian patients. Moreover, although factors such as ethnicity, low educational level, older age, chemotherapy and heavy work have been negatively associated with return to work, it is unknown if these factors apply in a cancer rehabilitation setting [14].

Hence, our objective was to investigate the RTW outcomes of breast cancer patients undergoing a community-based RTW rehabilitation program in a cancer rehabilitation center. An additional objective was to investigate the factors associated with RTW in this population.

## 2. Materials and Methods

### 2.1. Study Design

This was a retrospective cohort pilot study of breast cancer patients who had presented at a national community-based cancer rehabilitation center and enrolled in a RTW rehabilitation program from Jan. 2018 to Sep. 2019. Cancer patients were referred from clinicians from local healthcare institutions or from primary care following completion of acute cancer treatment.

### 2.2. Patients

Eligible patients for this study were breast cancer patients, aged 21–65 years old, who had been in paid employment prior to cancer diagnosis and were treated and were enrolled in the RTW rehabilitation program. Patients were excluded if they had retired, had severe cognitive or physical impairment limiting rehabilitation or refused to participate. Sociodemographic and clinical information were obtained from medical records. The stage of breast cancer was based on the American Joint Committee on Cancer (AJCC) staging system [15]. This clinical study adhered to the principles of the Declaration of Helsinki. The study was approved by the local institutional ethics committee—Agency for Integrated Care Institutional Review Board (2020-009).

### 2.3. Return to Work Program

All patients first attended an initial assessment visit, where they would be assessed by a physiatrist, physical therapist and occupational therapist. Based on medical suitability, patients would then be invited to enroll in the RTW rehabilitation program.

The RTW rehabilitation program is a community-based outpatient interdisciplinary vocational rehabilitation program established by the Singapore Cancer Society Rehabilitation Center, as part of the comprehensive rehabilitation services offered there [16]. The overall goals of the RTW rehabilitation program were to improve the work and quality of life of the participants through interdisciplinary rehabilitation.

Key components of the program included physical exercise, use of assistive technology, pain control, psychosocial support, work resumption advice and energy conservation strategies. Participants were also provided advice on cancer treatment, side effects and coping strategies. These services were individualized to the needs of each participant, and delivered by an interdisciplinary team of physiatrists, occupational therapists, physiotherapists and social workers. Each participant’s progress was routinely reviewed at interdisciplinary meetings. Participants were discharged when they had returned to sustained employment. Further details of the program are in Table 1.

Fatigue prior to enrollment in rehabilitation was evaluated using the Brief Fatigue Inventory (BFI), which is widely used for screening of fatigue severity in cancer patients [17]. The BFI comprises 9 items on fatigue which rate severity on an 11-point scale from 0 (no fatigue) to 10 (as bad as you can imagine). The initial 3 items ask patients to describe their fatigue now, at its usual level, and at its worst level during the previous 24 h. The next 6 items ask patients to describe how much fatigue has interfered with different aspects of their life during the previous 24 h from 0 (does not interfere) to 10 (completely interferes), in the areas of general activity, mood, walking ability, normal work, relations with other people and enjoyment of life. The global score for the BFI is derived from the mean value of these 9 items, with clinically significant BFI scores defined as ≥4 [17]. Good internal consistency has been reported with Cronbach alphas of 0.96 for fatigue-related severity and 0.95 for interference, with test-retest reliability being 0.89 for fatigue severity and 0.91 for interference [18].

Perceived work ability was evaluated with the Work Ability Index (WAI) [19]. This is a participant-reported measure of current work ability, compared with the lifetime best, which asks the question: “Assume that your work ability at its best has a value of 10 points. What score would you give your current work ability?” This is graded on a 11-point scale from 0 to 10, which was then categorized into as low (0–5) or adequate (6–10) [20]. Current perceived work ability is a reliable and valid indicator of work ability [21,22], and has the highest discriminating power in the entire index [23]. It is also associated strongly with the whole Work Ability Index [24,25].

### 2.4. Outcome Measures

The primary outcome measure studied was RTW as a dichotomous outcome, which was defined as any work resumption for at least 6 months.

We also obtained information on the date of work entry, occupation type (blue collar or white collar) and whether it was a part- or full-time job.

### 2.5. Statistical Analysis

Continuous variables with normal distribution were presented as mean and standard deviation, while those with non-parametric distribution were presented as median and 25–75th percentiles as a measure of position. Frequencies and percentages were used to present categorical variables. Normality was determined by the Kolmogorov–Smirnov test [26]. The distribution of categorical and continuous variables was compared using chi-squared test and the independent T-test, respectively. Paired data were analyzed with paired-sample T-test or McNemar test for continuously and categorical variables. To analyze the association between clinical variables and RTW, we performed univariate analyses, with the covariates of age, ethnicity, education level, previous job type, previous job working hours, cancer stage, cancer treatment, presence of clinically significant fatigue on BFI and perceived work ability on WAI at baseline. Significant factors on univariate analysis were then adjusted for in a multivariable logistic regression model. SPSS version 26.0 (IBM Corp., Armonk, NY, USA) software was used for statistical analysis. Statistical significance was set at *p* ≤ 0.05. Given a sample size of 63 patients, the expected effect size is approximately 1.03 at 80% power, α = 0.05.

## 3. Results

There was a total of 63 patients with breast cancer who were recruited into the RTW rehabilitation program. There were two patients excluded due to death (2), while two patients stopped rehabilitation due to cancer recurrence resulting in physical and cognitive deterioration. There were 41 (65.1%) patients with a cancer stage of 1–2 (Table 2).

A large proportion of patients had clinically significant fatigue levels on the BFI (36.5%) at baseline. The study population had a mean score of 2.14 (SD = 0.74) on the perceived work ability at baseline prior upon enrolment into the RTW rehabilitation program. After the program, there were significantly fewer patients with clinically significant fatigue levels (11.1%, *p* = 0.001) and an improved perceived work ability on the WAI with a mean score of 4.54 (SD = 0.77, *p* < 0.001).

After undergoing the RTW rehabilitation program, there were 37 (58.7%) participants who successfully returned to work. These participants returned to work at either within 6 months (27.0%), 6–12 months (29.7%) or 12–24 months (43.2%) after enrollment into the program (Table 3).

On univariate analyses, positive associations with RTW were education of secondary level (Odds ratio [OR] = 6.07; 95% confidence interval [CI] = 1.42–26.0; *p* = 0.015) or tertiary level (OR = 4.44; 95% CI = 1.08–18.4; *p* = 0.039), working full time in their prior job (OR = 2.68; 95% CI = 1.93–3.73; *p* = 0.025) and a higher perceived work ability on WAI (OR = 3.58; 95% CI = 1.48–8.66; *p* = 0.005). Negative associations on univariate analyses with RTW were cancer stage of 3–4 (OR = 0.121; 95% CI = 0.037–0.393; *p* < 0.001), radiotherapy (OR = 0.280; 95% CI = 0.087–0.903; *p* = 0.033) and presence of clinically significant fatigue on BFI (OR = 0.069; 95% CI = 0.020–0.244; *p* < 0.001) (Table 4).

In the multivariate regression model, negative factors for RTW were advanced stage of cancer (OR = 0.125; 95% CI = 0.030–0.51; *p* = 0.004), along with clinically significant fatigue on BFI (OR = 0.064; 95% CI = 0.014–0.30; *p* < 0.001). However, perceived work ability on WAI (OR = 4.16; 95% CI = 1.25–13.82; *p* = 0.020) was found to be a positive factor for RTW (Table 5).

## 4. Discussion

Our study reports a RTW success rate of 58.7% in breast cancer patients after undergoing a multidisciplinary RTW rehabilitation program. A systemic review by Islam et al. found a prevalence of RTW ranging between 43% to 93% within 1 year of diagnosis [14], although most of these patients did not receive any structured RTW rehabilitation. There have been studies highlighting the success of rehabilitation in enabling RTW [27,28], which may have enabled a relatively high RTW success in our population. Addressing physical symptom burden through rehabilitation reduces work disability [29], and may also increase self confidence in physical ability [30]. Supporting the need for rehabilitation are the findings of a Cochrane review looking at 1835 patients with various cancers, which found moderate quality evidence that multi-disciplinary interventions, including physical, psycho-educational and/or vocational components, were effective in improving RTW rates compared to usual care [31].

Our study shows the importance of a rehabilitation-based program to improve the functional status of patients. For example, vocational counseling and rehabilitation services offer specialized support in navigating the RTW process, addressing vocational barriers and optimizing workplace accommodations and job modifications. For example, occupational therapy and physical therapy interventions focus on adaptive strategies, assistive devices and environmental modifications to facilitate task performance, optimize energy conservation and minimize disability in the context of advanced cancer. By integrating functional skill training, healthcare providers can enable patients to maintain work roles amidst the challenges of illness and debilitation.

Although even higher rates of RTW success have been reported in intervention programs for breast cancer patients [32], differences exist between those study settings and our patient population. It is likely that different welfare systems and labor laws have varying impacts on long term sick leave and disability policies [33,34], resulting in different RTW rates among countries. For example, the United Kingdom and Norway have universal health coverage, whereas the United States have both public and private health insurance coverage [32]. Additionally, given our study population of breast cancer patients, we speculate that there may be lower participation in the workplace by women in an Asian country, due to increased family commitments or sedentary behavior [35]. This is supported by a study in South Korea which had a relatively low rate of RTW of 37.1% within 3 years in breast cancer patients [34].

The study findings highlight the need for a structured and individualized approach in preparing breast cancer patients for return to employment. RTW can be challenging due to the presence of multiple physical and mental impairments related to physical disability, cancer itself and the treatment [36]. Although many studies report RTW outcomes at 1 year [31], we found that 43.2% of our study participants returned to work between 1 to 2 years after starting the RTW rehabilitation program. This suggests that such RTW programs may have to factor in a prolonged period of multidisciplinary rehabilitation to overcome these impairments. Studies with a follow-up duration of only several months may also be underestimating the potential for patients to RTW. This is supported by other studies with a follow-up duration of more than 12 months, which have demonstrated that a substantial percentage of cancer patients re-enter the workforce at 36 months post-treatment [34,37].

Additionally, despite interdisciplinary rehabilitation, we found that 27% of breast cancer patients who successfully returned to work required a change to part-time working hours. This highlights the need for flexibility in the working hours, as these patients may not have been able to regain full physical and cognitive function, and would benefit from work adjustments [38,39].

We also wish to highlight the role of cancer stage, fatigue and WAI scores in successful RTW. Advanced cancer is characterized by extensive tumor spread, metastasis to distant organs and often a higher tumor burden. Patients with advanced stage of cancer often have increased symptomatology, exacerbated treatment side effects and heightened overall debilitation. Patients with advanced cancer report higher levels of cancer related fatigue, pain and compromised organ function, which poses formidable barriers to the resumption of normal work activities [40,41,42]. Additionally, the nature of treatments required for advanced stages, such as intensified or multiple cycles of chemotherapy regimens and invasive surgeries, further extends recovery times, contributing to a prolonged period of functional impairment. Cumulative toxicities from aggressive treatments contribute to a higher prevalence of long-term side effects, ranging from chronic pain and neuropathy to cardiovascular and pulmonary complications [43]. Hence, these patients face a prolonged and challenging recovery period marked by persistent physical and cognitive impairment, which can hinder successful RTW.

Reported fatigue was high in this study, with more than a third of the participants reporting clinically significant fatigue on the BFI, which was additionally found to have a significant negative association with RTW on multivariate analysis. It is well-established that fatigue is a particularly common complication experienced by breast cancer patients and is a considerable barrier for successful RTW [44,45]. Various factors, such as physical inactivity, obesity and aromatase inhibitors, have been found to be associated with long-term cognitive and physical fatigue [46]. Our finding reinforces the importance of rehabilitative interventions, which have been proven to be effective in reducing fatigue, increasing cognition functioning and improving work performance [47]. Aerobic and multimodal exercises, which were a major component of our RTW rehabilitation program, are thought to combat cancer-related fatigue by improving aerobic capacity, muscular strength and endurance, flexibility and body composition. We believe that these physiological benefits synergistically contributed to fatigue reduction in cancer patients and enabled a successful RTW [48].

We also found that perceived work ability at baseline, assessed via the WAI, had a significant positive association with successful RTW. This indicates that perceived work ability is an important independent factor for the RTW process in breast cancer patients. A cancer patient’s cognitive representation of illness may influence their coping strategy. This suggests that, irrespective of impairments, the perception a cancer patient has about his or her work ability can also be a facilitator or barrier towards RTW [31].

## 5. Limitations

This study has several limitations that should be highlighted. Firstly, the exploratory and retrospective nature of this pilot study meant that there was a small sample size with an underpowered study design and lack of a control group, which limits generalizability and interpretation of results. We are also unable to determine the demographics, clinical factors and RTW rate of patients who have not enrolled in a rehabilitation program. Second, we were unable to obtain data on socioeconomic factors (e.g., household income, presence of children, social support), psychological factors (e.g., anxiety, depression) and detailed work-related factors (e.g., type of job, job facility, flexibility, support from colleagues and employers, duration of sick leave), which may affect RTW. Third, although perceived work ability was found to be a significant factor for RTW, further investigation into the various subscales of WAI and psychological factors (e.g., emotional states, fear of environmental hazards, self-motivation) will be useful to determine the subcomponents influencing perceived work ability.

## 6. Conclusions

In summary, we report a successful RTW rate of nearly half of all breast cancer patients enrolled in an interdisciplinary, outpatient-based RTW rehabilitation program. Our findings emphasize the key role of cancer rehabilitation in improving RTW rates in breast cancer patients. Further studies are required to determine the optimal duration of rehabilitation and type of interventions to address identified barriers so as to reduce exclusion of breast cancer patients from the job market. Clinicians also play an important role in detecting at-risk patients, especially those with advanced cancers, high levels of fatigue and low perceived work ability, such that these patients can receive suitable interventions early in the RTW process.

## Figures and Tables

**Table 1:** Details of the return to work rehabilitation program.

Parameters	Details
Frequency	Typically fortnightly or monthly
Duration of each session	30 min
Healthcare professionals involved	Physiatrists, physical therapists, occupational therapists, social workers
Components	Compensatory strategies, structured aerobic and resistance exercise program, workplace accessibility, pain control, functional skill training, assistive technology, mobility aids, transport
Mode of delivery	Individual

**Table 2:** Characteristics of the study population (N = 63).

Characteristics	n (%)/Mean ± SD
Age, *n* (%)	
≤60	46 (73.0)
>60	17 (27.0)
Ethnicity, *n* (%)	
- Chinese	56 (88.9)
- Malay	7 (11.1)
Breadwinner, *n* (%)	
- Sole	22 (34.9)
- Shared	41 (65.1)
Education level, *n* (%)	
- Primary	14 (22.2)
- Secondary	24 (38.1)
- Tertiary	25 (39.7)
Previous job, *n* (%)	
- Blue collar	16 (25.4)
- White collar	47 (74.6)
Previous job work hours, *n* (%)	
- Part time	4 (6.3)
- Full time	59 (93.7)
Cancer stage, *n* (%)	
- 1–2	41 (65.1)
- 3–4	22 (34.9)
Treatment, *n* (%)	
- Surgery	54 (85.7)
- Chemotherapy	50 (79.4)
- Radiation therapy	41 (65.1)
- Hormone therapy	33 (52.4)
BFI of ≥4, *n* (%)	23 (36.5)
Perceived work ability on WAI at baseline, mean ± SD	2.14 ± 0.74
Average number of physiotherapy sessions, mean ± SD	5.10 ± 5.06
Average number of occupational therapy sessions, mean ± SD	7.00 ± 4.85

WAI: Work Ability Index.

**Table 3:** Clinical outcomes for participants who were successful in returning to work (N = 37).

Clinical Outcomes	*n* (%)
Date of work entry, *n* (%)	
- 6 months	10 (27.0)
- 6–12 months	11 (29.7)
- 12–24 months	16 (43.2)
Change of working hours from full time to part time, *n* (%)	10 (27.0)
Type of job, *n* (%)	
- Blue collar	5 (13.5)
- White collar	32 (86.5)
New job work hours, *n* (%)	
- Part time	10 (27.0)
- Full time	27 (73.0)

**Table 4:** Univariate analysis of associations with successful return to work.

Characteristics	Univariate Analysis	
	Odds Ratio (95% CI)	*p*-Value
Age		
- <=60 years	1.0	
- >60 years	0.37 (0.12–1.17)	0.085
Ethnicity		
- Chinese	1.0	
- Malay	0.929 (0.19–4.55)	0.928
Education level		
- Primary	1.0	
- Secondary	6.07 (1.42–26.0)	0.015
- Tertiary	4.44 (1.08–18.4)	0.039
Previous job		
- Blue collar	1.0	
- White collar	3.23 (0.99–10.49)	0.051
Previous job work hours		
- Part time	1.0	
- Full time	2.68 (1.93–3.73)	0.025
Cancer stage		
- 1–2	1.0	
- 3–4	0.121 (0.037–0.393)	<0.001
Treatment		
- Surgery	1.16 (0.28–4.83)	0.835
- Chemotherapy	0.863 (0.247–3.014)	0.817
- Radiotherapy	0.280 (0.087–0.903)	0.033
- Hormonal therapy	0.905 (0.331–2.47)	0.845
Brief Fatigue Inventory ≥4		
<4	1.0	
≥4	0.069 (0.020–0.244)	<0.001
Perceived work ability on WAI	3.58 (1.48–8.66)	0.005

WAI: Work Ability Index.

**Table 5:** Multivariate analysis of associations with successful return to work.

Characteristics	Multivariate Analysis	
	Odds Ratio (95% CI)	*p*-Value
Cancer stage		
- 1–2	1.0	
- 3–4	0.125 (0.030–0.51)	0.004
Brief Fatigue Inventory		
- <4	1.0	
- >=4	0.064 (0.014–0.30)	<0.001
Perceived work ability on WAI	4.16 (1.25–13.82)	0.020

WAI: Work Ability Index.

## Data Availability

The data presented in this study are available on request from the corresponding author.
